# PACSIN proteins in vivo: Roles in development and physiology

**DOI:** 10.1111/apha.13783

**Published:** 2022-01-20

**Authors:** Vincent Dumont, Sanna Lehtonen

**Affiliations:** ^1^ Department of Pathology and Research Program for Clinical and Molecular Metabolism Faculty of Medicine University of Helsinki Helsinki Finland; ^2^ Department of Pathology University of Helsinki Helsinki Finland

**Keywords:** actin cytoskeleton, BAR domain, endocytosis, membrane shaping, PACSIN, syndapin, trafficking

## Abstract

Protein kinase C and casein kinase substrate in neurons (PACSINs), or syndapins (synaptic dynamin‐associated proteins), are a family of proteins involved in the regulation of cell cytoskeleton, intracellular trafficking and signalling. Over the last twenty years, PACSINs have been mostly studied in the in vitro and ex vivo settings, and only in the last decade reports on their function in vivo have emerged. We first summarize the identification, structure and cellular functions of PACSINs, and then focus on the relevance of PACSINs in vivo. During development in various model organisms, PACSINs participate in diverse processes, such as neural crest cell development, gastrulation, laterality development and neuromuscular junction formation. In mouse, PACSIN2 regulates angiogenesis during retinal development and in human, PACSIN2 associates with monosomy and embryonic implantation. In adulthood, PACSIN1 has been extensively studied in the brain and shown to regulate neuromorphogenesis, receptor trafficking and synaptic plasticity. Several genetic studies suggest a role for PACSIN1 in the development of schizophrenia, which is also supported by the phenotype of mice depleted of PACSIN1. PACSIN2 plays an essential role in the maintenance of intestinal homeostasis and participates in kidney repair processes after injury. PACSIN3 is abundant in muscle tissue and necessary for caveolar biogenesis to create membrane reservoirs, thus controlling muscle function, and has been linked to certain genetic muscular disorders. The above examples illustrate the importance of PACSINs in diverse physiological or tissue repair processes in various organs, and associations to diseases when their functions are disturbed.

## 1 GENERAL ON PACSINS

### 1.1 Identification and structure

**FIGURE 1:**
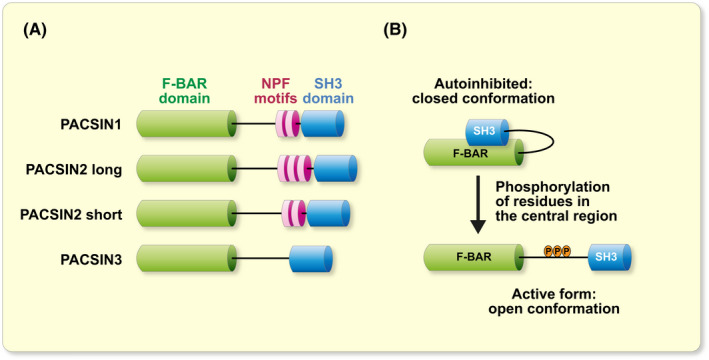
Structure of PACSINs and their autoinhibition. A, schematic view of PACSIN isoforms in mammals: PACSIN1, PACSIN2 long, PACSIN2 short and PACSIN3. B, cartoon of the release of the autoinhibition of PACSINs by phosphorylation of residues in their central region

The origin of PACSIN/syndapin dates back to year 1997 and identification of a novel focal adhesion protein FAP52 from chicken.^1^ A year later, a sequence highly homologous to FAP52 was described in mouse and named PACSIN,^2^ followed by identification of the rat homolog syndapin.^3^ For vertebrates, we will use the name PACSIN throughout this review, independent of the species, for simplicity. The following studies by Plomann and Qualmann characterized the structure and tissue distribution of this new protein family in mammals, comprising three members, PACSIN1, PACSIN2 and PACSIN3. All three PACSINs are composed of a Fes‐CIP4 homology Bin‐Amphiphysin‐Rvs161/167 (F‐BAR) domain in the N‐terminal region, a Src‐homology 3 (SH3) domain in the C‐terminal region and a variable central region that includes asparagine‐proline‐phenylalanine (NPF) motifs in the case of PACSIN1 and PACSIN2.^2^, ^3^, ^4^, ^5^, ^6^, ^7^ Of interest, short and long forms of PACSIN2 can be expressed in various cell types and tissues, with a difference in the number of NPF motifs^6^ (Figure 1A).

The F‐BAR domain is conserved amongst PACSIN proteins and is a subclass of the larger BAR domain family, known to associate with the negatively‐charged residues of cellular membranes.^5^, ^8^, ^9^, ^10^ The F‐BAR domains have a concave, banana‐like tertiary structure that can sense and induce curvature to lipid bilayers.^8^ PACSINs can also oligomerize via their F‐BAR domain, forming necks around membrane invaginations, such as caveolae and clathrin‐coated pits.^5^, ^10^, ^11^, ^12^ The SH3 domain and NPF motifs mediate the interaction of PACSINs with other proteins involved in endocytosis, regulation of the cytoskeleton, signalling and receptor trafficking (see Table 1 for details and references). Importantly, the activity of PACSINs can be regulated by autoinhibition when their SH3 domain interacts with their F‐BAR domain.^12^, ^13^, ^14^, ^15^ In this case, PACSINs are in a closed conformation that can be opened by interaction of their SH3 domain with either dynamin or caveolin‐1 in the case of PACSIN1 or PACSIN2, respectively^13^, ^14^, ^15^ (Figure 1B). In the case of PACSIN1, the interaction of the SH3 domain with the proline‐rich domain of dynamin is stimulated by the dephosphorylation of specific residues of dynamin.^16^

**TABLE 1:** List of proteins known to interact or function with PACSINs in various cellular processes

Protein name	Function of the protein	PACSIN isoform	Domain involved*	References
Part A: proteins involved in intracellular trafficking				
Caveolin‐1	Caveolar coat protein	PACSIN1‐3	F‐BAR	15
Caveolin‐3	Caveolar coat protein	PACSIN3	F‐BAR	17
Dynamins	Membrane fission	PACSIN1‐3	SH3	3, 5, 6
Eps15 homology domain proteins	Membrane fission	PACSIN1/2	NPF	18
Huntingtin	Vesicular trafficking	PACSIN1	SH3	19
Inositol polyphosphate phosphatase interacting protein of 27 kDa A	Endosomal trafficking	PACSIN1‐3	SH3	20
MICAL‐L1	Endosomal trafficking	PACSIN2	SH3	21
PICK1	Adaptor protein involved in trafficking	PACSIN1‐3	central region	22
Rabenosyn‐5	Endosomal trafficking	PACSIN2	N/D	23
Synapsin	Synaptic vesicle trafficking	PACSIN1/2	SH3	3, 6
Synaptojanin	Clathrin‐mediated endocytosis	PACSIN1‐3	SH3	3, 5, 6
Part B: proteins involved in cytoskeleton regulation				
Actin	Actin cytoskeleton regulation	PACSIN2	F‐BAR	24
Alpha‐tubulin	Microtubule network protein	PACSIN1‐3	central region	25
Cordon Bleu	Actin cytoskeleton regulation	PACSIN1‐3	SH3	26
Filamin A	Actin cytoskeleton regulation	fap52, PACSIN2	F‐BAR	27, 28, 29
Gamma‐tubulin	Ciliogenesis	PACSIN1‐3	N/D	25
n‐wasp	Actin cytoskeleton regulation	PACSIN1‐3	SH3	3, 5, 6
Rac1	Actin cytoskeleton regulation	PACSIN1/2	SH3	30, 31
SPIN90	Actin cytoskeleton regulation	PACSIN1/2	SH3	32
Tau	Microtubule network protein	PACSIN1	SH3	33
Part C: proteins in complex with PACSINs				
ADAM 9, 10, 12, 15, 19	Metalloendopeptidases	PACSIN3	N/D	34
ADAM 13	Metalloendopeptidase	PACSIN2	SH3	35
Anillin	Cytokinesis	syndapin (*D. melanogaster*)	SH3	36
CD95L = fas ligand	Apoptosis	PACSIN2/3	SH3	37
CPj0678	Cell contact‐dependent type III secretion system	PACSIN2	N/D	38
Cyclin D1	Cell cycle	PACSIN2	NPF	39
Gag proteins (HIV and RSV)	Virus release	PACSIN2	N/D	40
GluA2	Neurotransmission	PACSIN1‐3	N/D	22
Glycine receptor beta subunit	Neurotransmission	PACSIN1/2	SH3	41
Itch	Protein ubiquitination	PACSIN1	SH3	42
mSos	Guanine nucleotide exchange factor	PACSIN1/2	SH3	43
Nonstructural Protein 5A	Replication, assembly, and release of hepatitis C virus	PACSIN2	F‐BAR	44
NR3A	Neurotransmission	PACSIN1	NPF	45
Phosphodiesterase 6 gamma	cGMP signalling	PACSIN1	SH3	46
Polycystin‐1	Calcium‐permeable ion channel	PACSIN2	F‐BAR	47
Potassium‐chloride transporter member 5 (KCC2)	Potassium‐chloride cotransport	PACSIN1	central region	48
ProSAP1/Shank2	Neurotransmission	PACSIN1‐3	SH3	49
ProSAP2/Shank3	Neurotransmission	PACSIN1	SH3	49
TRPV4	Non‐selective calcium channel	PACSIN1‐3	SH3	50
Part D: proteins whose trafficking is altered by PACSINs, interactions not studied				
Cation‐independent mannose 6‐phosphate receptor	Lysosomal protein trafficking	PACSIN2	N/D	20
Clostridium difficile Toxin A	Bacterial toxin	PACSIN2	N/D	51
EGF receptor	EFG signalling	PACSIN2	N/D	52
Glucose transporter 1	Glucose transport	PACSIN3	N/D	53
Nephrin	Renal function and insulin secretion	PACSIN2	N/D	23
Transferrin receptor	Iron uptake	PACSIN1‐3	N/D	5, 6, 54
VE‐cadherin	Adhesion of vascular epithelial cells	PACSIN2	N/D	55

Abbreviation: N/D, not determined.

* The domain of PACSIN involved, shown at least for one PACSIN isoform.

### 1.2 Expression and nomenclature in the tree of life

In the different taxa of the tree of life, the numbers of PACSINs vary. Edeling and colleagues published a dendrogram of the PACSIN family showing the number of PACSIN members in different species, including several model organisms.^56^ In non‐vertebrate species, such as *Caenorhabditis elegans* (nematode), *Drosophila melanogaster* (fruit fly), *Anopheles gambiae* (mosquito) and *Ciona intestinalis* (an ascidian species), only one member of PACSINs is present, called sndp‐1 (*C. elegans*), syndapin (*D. melanogaster* and *A. gambiae*) or PACSIN (*C. intestinalis*). In vertebrates, three paralog PACSIN genes are present, coding for PACSIN1‐3 (or syndapin 1‐3) proteins. This includes mammals, birds and amphibians. *Danio rerio* (zebrafish) has six PACSIN paralogs due to its genome duplication.^56^, ^57^

In mammals, the original studies indicate that PACSIN1 is neuron‐specific, with a strong expression in different parts of the brain, including the hippocampus and cerebellum.^2^ PACSIN2 is ubiquitously expressed, whereas PACSIN3 is found in skeletal muscle, heart and lungs.^2^, ^3^, ^4^, ^5^, ^6^, ^7^ However, several articles, including those on mammals, have simply reported “PACSIN”. Where appropriate, we changed PACSIN to PACSIN1‐3 when it was possible to identify which PACSIN isoform was studied based on methods or other articles by the same authors.

## 2 CELLULAR FUNCTIONS OF PACSINS

**FIGURE 2:**
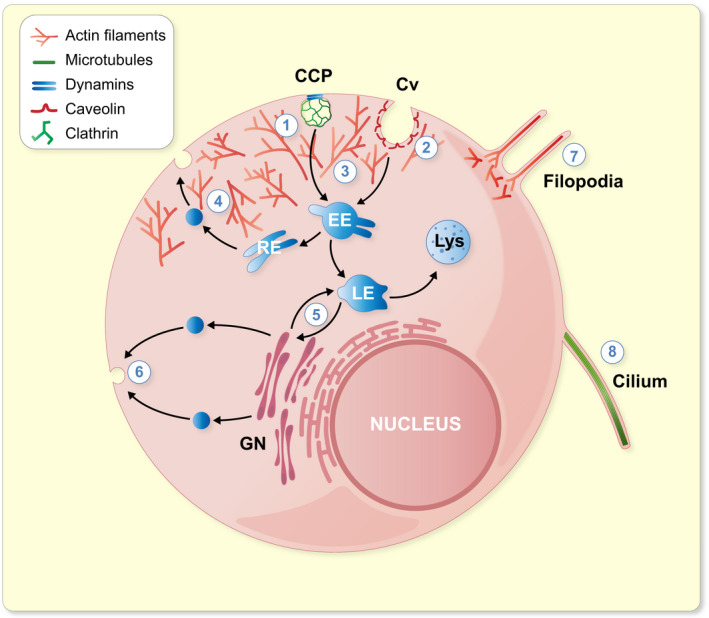
Intracellular functions regulated by PACSINs. 1: Regulation of clathrin‐dependent endocytosis with dynamins by pinching off the clathrin‐coated vesicles from the plasma membrane. 2: Biogenesis, stability and endocytosis of caveolae. 3: Regulation of cortical actin rearrangements necessary for vesicular trafficking. 4: Endosomal recycling. 5: Endosome‐to‐Golgi trafficking. 6: Exocytosis. 7: Filopodia formation. 8: Cilia formation. CCP, clathrin‐coated pit; Cv, caveola; EE, early endosome; GN, Golgi network; LE, late endosome; Lys, lysosome; RE, recycling endosome

PACSINs are well‐known regulators of the actin cytoskeleton and endocytosis.^5^, ^6^ The role of PACSINs in the vesicular trafficking has been refined to the regulation of clathrin‐dependent and independent endocytic pathways (CDE and CIE, respectively), and extended to endosomal recycling, endosome‐to‐Golgi trafficking and exocytosis.^15^, ^18^, ^20^, ^21^, ^58^, ^59^, ^60^, ^61^, ^62^, ^63^, ^64^, ^65^ In addition, a potential role for PACSINs in the regulation of cell cycle has been proposed and will be discussed in the “PACSINs in development” section. However, information about the precise functions of PACSIN1‐3 in the different intracellular compartments and different cell types, as well as the possible overlap between their functions remains poorly understood. Several excellent reviews cover these topics.^8^, ^10^, ^57^, ^66^, ^67^ Here, we will provide an overview of the functions of PACSINs defined by studies in vitro, summarized in Figure 2, before reviewing in more detail the studies that describe the roles of PACSINs in vivo.

### 2.1 Intracellular trafficking

The functions of PACSINs are best characterized in the brain. Early on, studies revealed that PACSIN1 is highly expressed in the neurons and plays a role in synaptic vesicle endocytosis (SVE), and that this function relies on the interaction of PACSIN1 with the master regulators of endocytosis, such as dynamins, synaptojanin or synapsin.^2^, ^3^, ^5^, ^6^, ^68^ The role of PACSIN2 is also rather well established in the regulation of caveolar biogenesis and endocytosis.^5^, ^6^, ^15^, ^58^, ^62^ In addition, PACSIN2 regulates several steps of intracellular trafficking, in particular endosomal and endosome‐to‐Golgi trafficking.^20^, ^21^, ^63^, ^69^ PACSIN3, in turn, has been less studied. It has been suggested to function in the caveolar‐based mechanoprotection of cells, and in the trafficking of transient receptor potential cation channel subfamily V member 4 (TRPV4) and glucose transporter 1.^17^, ^50^, ^53^

#### 2.1.1 Endocytosis

PACSINs have been associated with endocytosis of the transferrin receptor in early 2000s suggesting that PACSINs play a role in CDE.^5^, ^6^ In the brain, PACSIN1 participates in endocytic processes in the presynaptic compartment by regulating activity‐dependent bulk endocytosis (ADBE).^16^, ^70^ The interaction of PACSIN1 with dynamin is essential for this form of stimulation‐induced SVE.^71^ ADBE is characterized by large membrane foldings that participate in removing excess membrane quickly from synapses by the formation of endosome‐like structures and nonselective endocytosis.^72^ Importantly, PACSIN1 appears necessary for ADBE while having no effect on CDE.^16^, ^70^

PACSIN2 is present at the late stage of CDE when the nascent clathrin‐coated vesicle is pinched off of the plasma membrane.^61^ However, the best established function of PACSIN2 is in caveolae‐dependent endocytosis, as knockdown of PACSIN2 induces an alteration of the caveolar architecture.^15^, ^58^ Moreover, the phosphorylation status of PACSIN2 alters the stability of caveolae at the plasma membrane, in a mechanism controlled by cholesterol levels.^62^, ^73^ The absence of PACSIN3 in vivo induces changes in caveolar protein content and loss of deep caveolae in muscle tissue, suggesting a role in caveolar biogenesis.^17^ The function of PACSINs in caveolae is further supported by the interaction of PACSINs with Eps15‐homology domain containing (EHD) proteins, cavins and caveolins, involved in caveolar biogenesis and maintenance.^15^, ^17^, ^18^ These points are discussed in more detail in the “PACSINs in muscle biology” section. Beside the role in caveolar invagination, PACSIN3 is involved in the membrane channel trafficking. Specifically, it regulates the trafficking of glucose transporter 1 in adipocytes^53^ and TRPV4 in muscle cells.^50^ The latter is associated with a human genetic disorder.^74^, ^75^

#### 2.1.2 Endosomal recycling and endosome‐to‐Golgi trafficking

The interaction of PACSIN1 and PACSIN2 with EHD proteins implies a role for PACSINs in the endosomal trafficking and recycling.^18^, ^76^ PACSIN2, in particular, is known to cooperate with EHD proteins, MICAL‐L1 and OCRL1 to promote endosomal recycling from rabenosyn‐5 positive endosomes.^20^, ^21^, ^23^, ^63^, ^69^ In addition, PACSIN2 functions in endosome‐to‐Golgi trafficking and at the trans‐Golgi network.^20^, ^77^

#### 2.1.3 Exocytosis

Only few studies identify a role for PACSINs in exocytosis.^64^, ^65^ PACSIN1 and PACSIN2 have been observed at the sites of calcium‐induced exocytosis in endocrine cells,^64^ and suggested to function in the fusion pore expansion in chromaffin cells, a process involving active endo‐ and exocytosis.^65^ Furthermore, the regulation of the endosomal recycling by PACSINs suggests that PACSINs regulate the delivery of vesicles from intracellular stores to the plasma membrane. For example, PACSINs have been shown to participate in the recycling of epidermal growth factor (EGF) receptor, nephrin, cation‐independent mannose 6‐phosphate receptor and α‐amino‐3‐hydroxy‐5‐methyl‐4‐isoxazolepropionic acid (AMPA) receptor GluA2.^20^, ^22^, ^23^, ^52^, ^78^

Overall, PACSINs have been shown to regulate various steps of the intracellular trafficking of numerous proteins, channels and receptors. Table 1 provides examples of proteins that regulate the vesicular trafficking together with PACSINs.

### 2.2 Regulation of the cytoskeleton

The other well‐established function of PACSINs is the regulation of the actin cytoskeleton. Specifically, PACSINs have been proposed to link endocytic processes to the actin cytoskeleton.^3^, ^5^, ^6^, ^57^ All PACSINs interact with proteins that regulate the actin cytoskeleton, such as Neuronal‐Wiskott Aldrich Syndrome Protein (N‐WASP).^3^, ^5^, ^6^, ^57^ Overexpression of PACSIN1 or PACSIN2 induces filopodia formation in an N‐WASP‐Arp2/3 ‐dependent manner.^5^, ^6^ PACSIN1 also functions in the targeting of the actin nucleator Cordon Bleu (CoBl) to the plasma membrane, thereby regulating neuronal morphogenesis.^26^ Phosphorylation of PACSIN1 at serine 358 also regulates the function of rac1, well established as a regulator of the actin cytoskeleton that is associated with various diseases, such as developmental disorders and cancer.^30^, ^79^, ^80^ Recently, studies on PACSIN2 knockout mice revealed a role for PACSIN2 in the regulation of the actin cytoskeleton in vivo, as these mice presented altered actin structures at the apical brush border of enterocytes (see “PACSINs in intestinal biology” section).^81^

Although less studied, PACSINs also apparently regulate the microtubule network.^25^ All PACSINs can interact with alpha‐ and gamma‐tubulin, but appear to associate only with unpolymerized tubulin.^25^ Moreover, PACSIN2 knockdown by siRNA impairs microtube nucleation from centrosomes.^25^ The interaction of PACSIN1 with Tau also argues in favour of a role for PACSIN1 in the regulation of the microtubule network as Tau is known to polymerize microtubules.^33^, ^82^ PACSINs are also involved in the regulation of ciliogenesis^83^, ^84^ apparently via their association with gamma‐tubulin,^25^ and can thereby regulate organogenesis and kidney tubule repair after injury.^83^, ^84^ The role of PACSINs in ciliogenesis is discussed in more detail below.

## 3 PACSINS IN DEVELOPMENT

**FIGURE 3:**
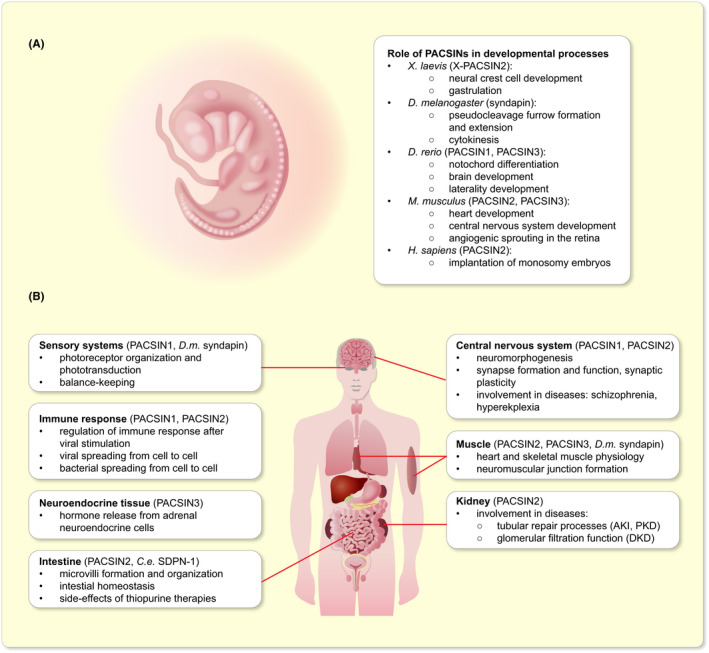
Summary of the main functions of PACSINs. A, Summary of the functions of PACSINs during development. B, Summary of the functions of PACSINs in various organs and organ systems in adulthood and in the regulation of the immune response. AKI, acute kidney injury; *C. e*., *Caenorhabditis elegans*; DKD, diabetic kidney disease; *D. m*., *Drosophila melanogaster*; PKD, polycystic kidney disease

Several studies have characterized the role of PACSINs in the development of vertebrate and non‐vertebrate species. Here, we will review the role of PACSINs in the development of *Xenopus laevis*, *D*. *melanogaster* and *D*. *rerio*, and also describe the potential role of PACSIN2 in monosomy in human. The role of PACSINs in the development of specific organs will be covered in the following sections and is summarized in Figure 3A.

### 3.1 Development of *X. laevis*

*X. laevis* is often used as a model organism for the development of vertebrates. A potential role for PACSINs in the development of *X. laevis* was revealed by the identification of the *X. laevis* homologue of PACSIN2 as an interaction partner of the cell surface matrix metalloprotease ADAM13.^35^ Overexpression of ADAM13 in *X. laevis* causes developmental defects, including extension of the mucus‐secreting cement gland that is involved in axial patterning, epidermal blistering and shortening of the anteroposterior axis.^35^ All these defects are rescued by overexpression of PACSIN2.^35^ Overexpression of a mutant form of PACSIN2, lacking the SH3 domain which mediates binding to ADAM13, appeared to increase the activity of ADAM13.^35^ This suggests that PACSIN2 negatively regulates the metalloprotease activity of ADAM13.^35^ Moreover, the overexpression of wildtype PACSIN2 in *X. laevis* leads to a mild defect in the neural crest cell development, whereas overexpression of PACSIN2 lacking its SH3 domain results in severe alterations, likely due to increased ADAM13 activity.^35^

Overexpression of wildtype PACSIN2 in *X. laevis* embryos inhibits gastrulation by a mechanism that depends on the coil‐coiled domain of PACSIN2, suggesting that the association of PACSIN2 with the plasma membrane is necessary for the completion of gastrulation.^27^ This associated with perturbed fibronectin fibrillogenesis and defective spreading of embryo‐derived ectodermal cells. The study also revealed that in cultured *X. laevis* XTC cells, overexpression of wildtype PACSIN2 alters the localization of integrin β1 and its binding partner filamin. Importantly, overexpression of mitochondria‐targeted PACSIN2, resulting in the withdrawal of endogenous PACSIN2 from the plasma membrane to the mitochondria, also blocks the gastrulation of *X. laevis*. This suggests that tight regulation of the activity of PACSIN2 and its presence at the plasma membrane is needed for the progression towards the late stage of gastrulation.^27^ Collectively, these studies indicate that PACSIN2 functions together with different binding partners at various stages of *X. laevis* development.

### 3.2 Development of *D. melanogaster*

The role of syndapin (the only protein of the PACSIN family in *D*. *melanogaster*) in the early *D*. *melanogaster* development is linked to its central activities in binding to negatively charged lipids and its ability to remodel membranes and the cytoskeleton. In the beginning of the development of *D. melanogaster*, the nuclei within the egg share a common cytoplasm.^85^ In this syncytial blastoderm‐stage embryo, the nuclei migrate to the periphery, and invaginations, called pseudocleavage furrows, originate from the cell surface and dynamically extend and regress between the dividing nuclei. The formation and regulation of the pseudocleavage furrows relies on several cellular machineries, including the central cytoskeletal components, namely actin, microtubules and septins, and small GTPases involved in intracellular trafficking. At this stage of development, syndapin localizes to the apical and lateral membranes of the pseudocleavage furrows and is further enriched in their tips.^86^ Syndapin is necessary for the recruitment and distribution of septin Peanut, formin Diaphanous and F‐actin to the pseudocleavage furrow, and mutating syndapin results in the disorganization of the furrow and decreased furrow extension.^86^ Interestingly, Rhogef2‐depleted embryos, presenting a defect in the pseudocleavage furrow formation similar to syndapin mutants, can be partially rescued by overexpression of syndapin.^86^

In a follow‐up study, Sherlekar et al^87^ studied the role of syndapin in apical actin cap remodelling in the syncytial blastoderm embryos. The apical Arp2/3‐based actin caps, encircled by actomyosin border, surround the peripheral nuclei in the syncytial embryo and control the pseudocleavage furrow extension. During the nuclear cycles, the apical cap expands and the furrow forms from the bending actin cap.^88^ The apical caps are larger in syndapin‐depleted embryos, and the apical actin protrusions of the caps remain constitutively long compared to control embryos, which have long protrusions during interphase and short ones during metaphase.^87^ Syndapin‐depleted embryos show defects in F‐actin dynamics, increased Arp2/3 activity as well as reduced myosin II expression and its sustained association with the pseudocleavage furrow.^87^ These studies reveal a central role for syndapin in the regulation of the pseudocleavage furrow extension in the developing *D. melanogaster* embryo.

Takeda et al^36^ studied the role of syndapin in the larval stages of *D. melanogaster* development. The authors show that syndapin localizes to the cleavage furrow of neuroblasts in the third instar larval brains, and is also found in the cleavage furrow in primary spermatocytes. They generated a hypomorphic syndapin mutant, resulting in low syndapin levels, and these flies die at the third instar larval stage indicating the necessity of syndapin for *D. melanogaster* development. The study also reported that the mutation induced multi‐nucleated spermatids revealing a central role for syndapin in cytokinesis during male meiosis.^36^ Expression studies of various syndapin constructs revealed that the localization of syndapin to the cleavage furrow is necessary to induce the defect in cytokinesis. Interestingly, the lipid‐binding F‐BAR domain alone is not sufficient for the recruitment of syndapin to the cleavage furrow, this requires also the adjacent 65‐residues in the linker region. Furthermore, the phosphorylation status of syndapin appeared important for the regulation of cytokinesis, as dephosphorylation of both the F‐BAR domain and the linker region was necessary for syndapin to localize to the cleavage furrow.^36^

Collectively, these studies show that the expression and phosphorylation of syndapin are of great importance in the early development of *D. melanogaster*.^36^, ^86^, ^87^

### 3.3 Development of *D. rerio*

Knockdown studies in *D. rerio* reveal essential roles for PACSIN1 and PACSIN3 in the early development.^56^, ^84^ In the developing *D. rerio*, PACSIN1 is expressed in neuronal tissues at 24 and 48 hours post fertilization (hpf).^84^ Morpholino antisense oligonucleotide (MO)‐driven knockdown of PACSIN1 led to shorter body length and curved body. The head and eyes of the morphants were smaller than those in the controls and they had heart and brain oedema, indicative of an impairment in brain development.^84^ The morphants had also laterality defects described more closely below in the paragraph on PACSINs in the sensory systems.

PACSIN3 functions in the differentiation of the notochord.^56^ Notochord is an embryonic midline structure with both signalling and structural functions necessary for the normal development of vertebrates.^89^ It secretes factors that control the fate and patterning of the surrounding cells and tissues and serves as a structural support in the form of primitive cartilage until the skeleton forms.^89^ PACSIN3 mRNA and protein were found to localize to notochord at 24 hpf.^56^ Knockdown of PACSIN3 with MO revealed severe developmental abnormalities in the embryos. Notochords failed to differentiate normally: the cells failed to intercalate, or form a single column, apparently due to a defect in cell migration, and the notochord showed spiral morphology. At 24 hpf, the MO‐injected embryos showed kinked bodies, shortened posteriors and altered somite organization indicating an essential role for PACSIN3 in the body patterning in *D. rerio*.^56^ Interestingly, the phenotype of the PACSIN3 MO‐treated *D. rerio* could be rescued by injection of MO‐resistant *D*. *melanogaster* syndapin mRNA indicating similar functions for PACSIN family members in *D. rerio* and *D. melanogaster*.^56^

### 3.4 Monosomy in human

An epigenetic study in human revealed reduced methylation on chromosome 22, involved in monosomy.^90^ Chromosome 22 also harbours PACSIN2 gene, locating within the highly hypomethylated cytogenic region. Gene expression analysis revealed lower expression level of PACSIN2 mRNA in monosomy blastocysts compared to controls.^90^ Monosomy embryos typically fail to implant, and the authors suggested, based on the studies in *X. laevis*,^27^ that decreased expression of PACSIN2 could reduce cell migration and thus the ability of the monosomy embryo to implant.^90^

## 4 PACSINS IN THE CENTRAL NERVOUS SYSTEM

PACSIN1 was originally identified by subtractive hybridization as a gene associated with self‐repair process in the mouse brain.^2^ Its expression was found to be developmentally regulated and to concentrate in the brain and dorsal root ganglia, with the highest level of expression in terminally differentiated neurons.^2^ In the years following its discovery, PACSIN1 has been extensively studied in cultured neurons, but its relevance in the central nervous system in vivo, as well as the function of PACSIN2, started to be clarified only in the past 10 years using knockout mice and proteomic and genetic studies.

### 4.1 Neuromorphogenesis

Koch and colleagues^91^ showed that mice lacking PACSIN1 display a reduction in the length of the dendritic tree of the cortical pyramidal neurons, and also their arborization and terminal points were reduced in specific layers of the cortex. Previous in vitro findings also revealed that PACSIN1 is involved in neuromorphogenesis by recruiting actin regulatory proteins to the plasma membrane to control actin filament formation.^26^, ^30^, ^92^ Specifically, PACSIN1 was found to regulate axon development together with N‐WASP^92^ and dendritic development with Cobl, an actin nucleator, also in complex with N‐WASP.^26^ Furthermore, phosphorylation of PACSIN1 on serine 358 by casein kinase is essential for dendritic spine formation.^30^ In line with the role of actin regulators in neuromorphogenesis in vitro, analysis of the brain revealed less membrane‐associated N‐WASP in mice that lack PACSIN1 when compared to wildtype mice, supporting that the PACSIN1‐N‐WASP complex is important for neuromorphogenesis also in vivo.^91^

### 4.2 Receptor trafficking and synaptic plasticity

Koch and colleagues^91^ further investigated the function of N‐methyl‐D‐aspartate (NMDA) and AMPA receptors in PACSIN1 deficient mice. NMDA and AMPA receptors (NMDAR and AMPAR, respectively) are widely expressed glutamate receptors in the brain and are found in the same synapses. GluA1 and GluA2 are subunits of AMPAR. AMPA and NMDA receptors regulate each other's activity via complex mechanisms that can lead to long term potentiation (LTP) or long‐term depression (LPD), both considered essential for synaptic plasticity.^93^

In the cultured primary hippocampal neurons of the PACSIN1 knockout mice, the number of GluA1 clusters in the synaptic areas was reduced while the overall density of GluA1 clusters did not differ between the neurons of the wildtype and knockout mice.^91^ The clusters were also smaller and the intensity of GluA1 in both synaptic and non‐synaptic clusters was reduced.^91^ Findings for GluA2 were similar in postsynaptic assemblies while no differences in the signal intensity of GluA2 was observed in the synaptic contact areas.^91^ PACSIN1 knockout mice also showed impaired AMPAR‐mediated evoked excitatory postsynaptic currents (eEPSCs) in CA1 pyramidal neurons.^91^ The authors argue that the increased diffuse pool of GluA1‐containing AMPARs at the plasma membrane results in hypofunction of AMPARs and lack of synchronicity required for the proper function of the synapse in the PACSIN1 knockout mice.^91^ These results are in line with a study showing that PACSIN1 knockdown impairs the NMDAR‐stimulated trafficking of AMPAR in hippocampal neurons, as shown by defects in the endocytosis and recycling of GluA2/3 subunits of AMPAR in a mechanism involving multiple structural domains of PACSIN1 and a phosphorylation‐dependent interaction of PACSIN1 and PICK1.^22^, ^78^ Contrary to knockout studies, overexpression of wildtype PACSIN1 or dominant‐negative PACSIN1 lacking the SH3 domain in cultured hippocampal neurons did not affect the surface expression of GluA1.^45^ However, the authors found that PACSIN1 regulates the endocytosis of NMDAR by removing the NR3A subunit from the plasma membrane.^45^ Supporting a role for PACSIN1 in AMPAR and NMDAR endocytosis, PACSIN1 knockout mice have enlarged synaptic vesicles in the hippocampus.^68^ Moreover, this study shows that PACSIN1 is needed for the recruitment of dynamin to lipid fractions of the plasma membrane, further arguing for a role in the regulation of endocytosis of synaptic vesicles in the hippocampal neurons.^68^

Since AMPARs and NMDARs regulate both LTP and LTD, the role of PACSIN1 has been examined in these processes. Both hippocampal LTP and LTD were reduced in PACSIN1 knockout mice, likely due to an imbalance in the presence of AMPAR and NMDAR at the plasma membrane.^91^ An earlier study revealed that the density of dendritic spines in the neurons of PACSIN1 knockout mice is reduced and that the postsynaptic functions of PACSIN1, in complex with postsynaptic protein ProSap1/Shank2, are essential for the formation of the synapse relying on PACSIN1‐enriched nanodomains acting as scaffolds.^49^ The study further revealed that the reduced frequency of excitatory miniature post‐synaptic currents in rat hippocampal neurons after knockdown of PACSIN1 was of postsynaptic origin.^49^ Reflecting back to the above‐described reduced availability of GluA1 at synapses in PACSIN1 deficient mice, Koch et al^91^ proposed that this could be partially due to altered localization of ProSap1/Shank2, as the clustering of receptors requires associations with postsynaptic density proteins. These data may explain the changes in the synaptic activity observed in the hippocampus of PACSIN1 knockout mice.^68^ In line with this, PACSIN2 is necessary for LTD in Purkinje cells of the cerebellum.^22^ Overall, these studies indicate that PACSIN1 and PACSIN2 may be important regulators of synapse formation and synaptic plasticity in different parts of the central nervous system, and that defects in these processes could explain the seizures observed in PACSIN1 knockout mice.^68^

### 4.3 PACSIN1 in the disorders of the central nervous system

#### 4.3.1 Schizophrenia

In 2008, Pennington and co‐workers found that PACSIN1 protein expression is reduced in the dorsolateral prefrontal cortex in patients with schizophrenia.^94^ An association between schizophrenia and a loci near PACSIN1 gene was found in several genetic studies, supporting a potential role for PACSIN1 in the development of schizophrenia.^95^, ^96^, ^97^ Moreover, an exome sequencing analysis of patients with schizophrenia revealed an ultra‐rare variant for PACSIN1 (R241Q).^98^

Importantly, Koch and colleagues^91^ confirmed that lack of PACSIN1 is involved in the appearance of schizophrenia‐like behaviour in mice. Specifically, PACSIN1 knockout mice displayed hyperactivity, reduced anxiety and reduced social interaction,^91^ all typical schizophrenia‐like behaviours in mice.^99^, ^100^ In addition, mice lacking PACSIN1 displayed more interest in new objects placed in the cage,^91^ similar to mice used as models for schizophrenia.^100^ In addition, the authors used in silico modelling of the R241Q variant of PACSIN1, and identified potential loss of intra‐ and intermolecular H‐bonds that could lead to functional defects.^91^ Indeed, the mutant showed altered membrane sculpting activity, and reduced interaction with dynamins, N‐WASP and CoBl. The defects hindered the normal function of PACSIN1 in neuromorphogenesis, as shown by the reduced length and arborization of the dendritic tree in primary neuronal cultures overexpressing PACSIN1‐R241Q compared to PACSIN1‐wildtype.^91^

A recent genetic study identified a polymorphism that associates with schizophrenia and the authors proposed that the polymorphism may regulate PACSIN3.^101^ These data require further studies on different patient cohorts and with larger sample size to confirm the result.

#### 4.3.2 Alzheimer's disease

PACSIN1 protein was found decreased in the hippocampus of a mouse model of Alzheimer's disease.^102^ The mice expressed a mutated form of amyloid precursor protein involved in the human disease. Interestingly, also clathrin light chain B was dysregulated in this model, suggesting an alteration of the endocytic machinery. This work further supports the importance of PACSIN1 in the hippocampal network activity.^102^

#### 4.3.3 Huntington's disease

PACSIN1, but not PACSIN2 or PACSIN3, has also been shown to interact with huntingtin, a protein involved in Huntington's disease.^19^ Analysis of a disease‐causing mutation in huntingtin revealed that the mutation leads to a higher association of huntingtin with PACSIN1. The authors suggest that the mutated form of huntingtin, localizing perinuclearly, could act as a sequester of PACSIN1, thus reducing the localization of PACSIN1 at the plasma membrane and hindering its normal functions. Supporting their hypothesis, immunostaining of presymptomatic patients with Huntington's disease indicate a relocation of PACSIN1 to the perinuclear area in neurons of the frontal cortex.^19^

#### 4.3.4 Startle disease

A recent study described PACSIN1 in association with startle disease or hyperekplexia, a neuromotor disorder caused by mutations in glycine receptors expressed in the spinal cord and brainstem.^103^ A novel mutation in glycine receptor subunit gene *GLRA1* was found to reduce binding of the receptor to PACSIN1.^103^ Furthermore, expression studies in hippocampal neurons revealed that the mutant receptor leads to an increase of PACSIN1 localization in the soma of the cells. The authors suggested that PACSIN1 could regulate the trafficking of the glycine receptor to the plasma membrane of the neurites and when this is defected due to their reduced complex formation, to contribute to the development of the disease.^103^

Overall, PACSIN1 appears as a protein whose function is essential for glutamatergic neurotransmission and efficient synaptic plasticity as well as the organization of the dendritic tree. PACSIN1 regulates the expression of NMDA and AMPA receptors on the cell surface either by directly interacting with the receptor or via regulatory molecules that bind to the receptor. Accordingly, PACSIN1 regulates synaptogenesis by directly interacting with the NR3 chain of the NMDA receptor and controls synaptic plasticity by regulating AMPA receptor trafficking via proteins such as PICK1. Defects in these processes appear as potential mechanisms leading to neurological disorders, including schizophrenia. The studies also suggest a role for PACSIN1 in various other neurological disorders but these will require further investigations for detailing the relevance in vivo.

## 5 PACSINS IN MUSCLE BIOLOGY

### 5.1 Heart development in mouse

In the developing mouse heart, PACSIN2 is expressed in both atrial and ventricular myocytes, and its depletion from mice leads to alterations in the cardiac function during development.^104^ Electrophysiological analysis of the whole hearts on embryonic day 15.5 revealed prolonged atrio‐ventricular conduction time in the knockout animals, associated with downregulated expression of the T‐box 3 transcription factor and gap junction protein connexin 43, both involved in cardiac conduction. Further analyses of action potentials using isolated foetal atriolar myocytes revealed that cells from the knockout mice beat at a lower frequency, with a longer depolarization time, than their wildtype counterparts. PACSIN2 deficiency also changed the current properties of several ion channels, including hyperpolarization‐activated cyclic nucleotide‐gated, sodium and L‐type calcium channels. Because loss of PACSIN2 caused only a moderate change in the electrophysiological properties of the ventricular myocytes, the authors suggest that PACSIN2 may play an important role in the regulation of the beating frequency of the developing heart by altering the atrial functions where the action potentials are initiated. The authors further propose that the alterations in the PACSIN2 knockout cardiomyocytes may result from improper targeting of ion channels to the plasma membrane.^104^

### 5.2 Heart and skeletal muscle physiology in adult mice

In the adult PACSIN2 knockout mice, many cardiac genes are downregulated in the heart tissue but despite this, the knockout mice display normal heart morphology.^104^ The authors mentioned, though, that the mice depleted of PACSIN2 show reduced running endurance, distance and speed compared to their wildtype counterparts.^104^ Another study reported that supplementing the diet with antioxidants, namely vitamin‐E and α‐lipoic acid, induces transcriptional upregulation of PACSIN2, among several other genes, in adult male rat myocardial tissue after 14 weeks of treatment.^105^ As the authors previously found that antioxidant supplementation improves cardiac performance, they suggested that an increase in PACSIN2 expression may be involved in the improvement of cardiac function by antioxidants.^105^

PACSIN3 is expressed in muscle tissue,^5^, ^57^ and its function in this tissue has been studied using knockout mice.^17^ In both cardiomyocytes and skeletal muscle, the absence of PACSIN3 results in a reduction of the number of deep membrane invaginations or caveolae. The overall immunolabelling density of caveolar coat protein caveolin‐3 at the plasma membrane did not differ between wildtype and PACSIN3 knockout cardiomyocytes, but the density of caveolin‐3 in the deeply invaginated caveolae reduced concomitant to their reduced number. Cavin1 is another caveolar coat protein and interestingly, PACSIN3 knockout in cardiomyocytes elevated the density of cavin1 at the plasma membrane, concentrating in large clusters which were often negative for caveolin‐3. Electron tomograms revealed that PACSIN3 is present at the neck of caveolin‐3 positive invaginations. Moreover, biochemical analysis of membrane preparations of heart and skeletal muscle tissues shows that PACSIN3 participates in the targeting of caveolin‐3 to specific membranous detergent‐resistant microdomains. Overall, these data indicate that PACSIN3 participates in defining the identity of caveolae and that caveolin‐3 and cavin1 alone are not enough to form caveolae but PACSIN3 is essential for caveolar invagination. Interestingly, cardiomyocytes from PACSIN3 knockout mice were more susceptible to hypotonic choc,^17^ in agreement with the concept that caveolae act as reservoirs of membrane,^106^ and that PACSIN3 is important for creating this reservoir. It is worth noting that the overall architecture of the heart tissue of trained and non‐trained mice is not affected by the absence of PACSIN3, although the number of caveolae is decreased. In skeletal muscle, however, the absence of PACSIN3 in trained mice resulted in the presence of hypertrophic and necrotic muscle fibres, a phenotype reminiscent of human myodystrophies resulting from mutations in the caveolin‐3 gene.^17^, ^107^, ^108^

A role for PACSIN3 in muscle biology is further supported by studies in high fat‐diet induced skeletal muscle atrophy in mice^109^ and work on primary rat myoblasts in vitro.^110^ In high fat‐diet fed mice, the authors found elevated phosphorylation of serine residues of PACSIN3 in skeletal muscle. The skeletal muscles of these mice present signs of atrophy, inflammation and activation of the protein degradation pathway.^109^ The authors discussed the potential effect of the phosphorylation of PACSIN3 as a means to inhibit its activity in glucose transport, but further studies are necessary to establish whether the phosphorylation of PACSIN3 plays a role in muscle degradation. In the in vitro study, incubation of differentiating myoblasts with IL‐6, known to be expressed in injured muscles and suspected to promote the injury, leads to the downregulation of PACSIN3 at protein level.^110^

Moreover, a collection of studies demonstrate that interaction of PACSIN3 with TRPV4 is necessary for the normal function of the channel.^111^, ^112^, ^113^ Notably, TRPV4 has been found mutated in various forms of neuromuscular disorders and skeletal dysplasias.^75^ A mutation in *TRPV4* was found near the PACSIN3‐binding site, suggesting that lack of an interaction with PACSIN3 may be involved in the development of certain forms of TRPV4‐associated muscular diseases.^74^

Overall, proteins of the PACSIN family appear to play a role in muscle biology and may participate in the pathophysiological processes of some genetic muscular diseases.

### 5.3 Neuromuscular junction formation in *D. melanogaster*

In *D. melanogaster*, syndapin mRNA is widely expressed in the early stages of embryogenesis,^114^ including the mesoderm from which all muscle cells develop.^115^ Homozygous syndapin null mutant embryos showed no defects in the embryonic muscle system.^115^ However, double or triple mutants of syndapin and F‐BAR proteins nostrin and Cip4 revealed mild muscular defects, the phenotypes suggesting that syndapin may function in cooperation with these two F‐BAR family members in myoblast fusion.^115^

In the larvae of *D. melanogaster*, syndapin regulates the formation of neuromuscular junctions,^116^ which connect the motor neurons to skeletal muscle fibres. Neuromuscular junctions are essentially organised as synapses, with a presynaptic neuron, a synaptic cleft and the postsynaptic membrane on the motor end plate of muscles. Contrary to expectations, syndapin was not observed in the presynaptic compartment and was found to be dispensable for synaptic vesicle endocytosis.^114^ Instead, syndapin accumulated in the tubulolamellar postsynaptic membrane network called subsynaptic reticulum, and its overexpression resulted in an increased volume of the subsynaptic reticulum network.^116^ This, however, did not alter the function of the synapses. Mapping the domains necessary for this activity by generating transgenic *D. melanogaster* lines indicated that the F‐BAR domain of syndapin alone is capable of tubulating membranes, but that targeting syndapin to the postsynapse requires both its F‐BAR and SH3 domains. Importantly, stimulation of the expansion of the subsynaptic reticulum network occurs independently of Dlg and dPAK, central signalling molecules involved in subsynaptic reticulum biogenesis, suggesting a direct role for syndapin.^116^ Further work in *D. melanogaster* and the *D. melanogaster* S2 cell line suggested that syndapin may function together with the EHD protein Past1 and another membrane remodelling protein Amphiphysin to sequentially modulate the complex structure of the subsynaptic reticulum network.^117^

## 6 PACSINS IN INTESTINAL BIOLOGY

### 6.1 Endosomal system of the intestinal endothelium of *C. elegans*

An important part of the in vivo literature investigating PACSINs demonstrate a role in the maintenance of the intestinal homeostasis via their roles in the regulation of the vesicle trafficking and actin cytoskeleton. *C. elegans* is an excellent in vivo model to study regulators of intestinal homeostasis and has been utilized in several studies defining the role of PACSINs in the intestine. The expression of GFP‐tagged SDPN‐1, the only PACSIN family member in *C*. *elegans*, using its own promoter revealed localization of SDPN‐1 in the intestine, where it concentrated on the early endosomes and basolateral recycling endosomes.^118^, ^119^ In the worm, the localization of SDPN‐1 appears to be regulated by several proteins involved in the recycling processes, including alx‐1, which is involved in the maturation of multivesicular bodies, and RME‐1, an ortholog of mammalian EHD‐1 in *C*. *elegans*, which functions in recycling.^120^, ^121^ The alx‐1 mutants accumulate recycling cargo, and interestingly, SDPN‐1 fails to localize to the recycling endosomes in these mutants. In RME‐1 mutants, the number of SDPN‐1 positive recycling endosomes is reduced, and the ones expressing SDPN‐1 are enlarged.^118^ The authors found that the recruitment of SDPN‐1 to the recycling endosomes requires an interaction between alx‐1 and RME‐1 and suggested that alx‐1 promotes recycling through SDPN‐1.^118^ This suggests that the recycling processes are blocked in alx‐1 and RME‐1 mutants, and that SDPN‐1 is unable to generate endosomal intermediates unless it can form the required protein complexes. Work on SDPN‐1 mutant, leading to the total absence of the functional SDPN‐1 protein, confirmed a central role for SDPN‐1 in cargo trafficking in the intestine.^119^ Notably, lack of SDPN‐1 altered the expression pattern of proteins specific for various endosomal compartments, leading to loss of their compartmental identity and defects in the basolateral recycling of cargo. As in mammalian cells, SDPN‐1 was found to recycle both clathrin‐dependent and ‐independent cargos in *C*. *elegans*.^119^

Further work on *C. elegans* revealed that F‐actin is enriched on SDPN‐1 positive endosomes, and that the early endosomes in the SDPN‐1 mutant are devoid of F‐actin enrichment.^119^ Based on the localization pattern of SDPN‐1 and the idea that polymerization of actin on endosomes is necessary for endosomal fission, the authors proposed that SDPN‐1 could promote the fission of early endosomal membranes that are acquiring the characteristics of recycling endosomes. In the absence of SDPN‐1, this process is perturbed leading to trapping of the recycling cargo.^119^ A recent study identified RTKN‐1/Rhotekin, an effector of the Rho GTPase, as a new molecule necessary for endosomal recycling via the regulation of endosome‐associated F‐actin structures in the intestine of *C. elegans*.^122^ Interestingly, targeting of RTKN‐1 to endosomes appears to depend on SDPN‐1, stressing out the importance of SDPN‐1 for the normal physiology of the intestine in worms via the intertwined functions of endosomal recycling and actin cytoskeleton.^122^

Another level of regulation for SDPN‐1 is brought by phosphoinositides, and specifically, phosphoinositide PI(4,5)P2. PI(4,5)P2 plays a central role in endosomal recycling^123^ and interestingly, in *C. elegans* with mutated Arf‐6 and CNT‐1, also known to regulate endosomal recycling, the expression of SDPN‐1 was altered in the intestinal basolateral recycling endosomes due to changed concentrations of PI(4,5)P2 in this location.^124^ This indicates that modulating the amounts of PI(4,5)P2 in the endosomal recycling compartment in vivo, and thus altering their ability to recruit SDPN‐1, plays a key role in regulating the trafficking processes and thereby controlling intestinal homeostasis.

### 6.2 Intestinal microvilli formation and endocytosis in the intestinal endothelium in mammals

A study using knockout mice reveals a role for PACSIN2 in the maintenance of the intestinal tract.^81^ In both mouse and human small intestine, PACSIN2 is expressed near the base of the microvilli, where it colocalizes with F‐actin and the actin filament nucleator CoBl,^125^, ^126^ which also interacts with PACSIN2.^26^, ^84^, ^126^, ^127^ The intestinal cells, or enterocytes, build up a dense network of brush border microvilli that are involved in nutrient absorption and host defence and thus essential for the intestinal function. The microvilli are supported by an actin bundle, and various proteins that regulate actin play key roles in microvilli formation. The enterocytes of the PACSIN2 knockout mice have less microvilli and the microvilli lack the typical hexagonal organization observed in normal enterocytes. The microvilli show also morphological alterations: they are shorter, the length of the actin core and its membrane coverage are reduced, and the microvillar rootlet is longer in the PACSIN2 knockout mice.^81^ The absence of PACSIN2 also leads to the reduced presence of actin at the apical domain of enterocytes, coupled with reduced expression of CoBl. Interestingly, lack of PACSIN2 reduces the presence of endocytic proteins Vamp4 and dynamin2 at the base of the brush border, leading to reduced endocytic capacity of enterocytes and the presence of stalled endocytic structures in the intermicrovillar regions. Studies on cultured enterocytes reveal that inhibitors of endocytosis and dynamin2 lead to a similar outcome.^81^ These data together suggest that alterations in the endocytosis machinery and actin cytoskeleton likely lead to intestinal brush border defects observed in the PACSIN2 knockout mice.

Studies in vitro reveal that overexpression of CoBl in epithelial cells leads to the accumulation of PACSIN2 at the basis of the microvillae.^126^ Further studies in cells show that knockdown of PACSIN2 reduces the ability of the cells to form brush border, coupled with loss of CoBl at the base of the microvilli,^125^ supporting the findings in PACSIN2 knockout mice. These data suggest that PACSIN2 participates in the regulation of actin cytoskeleton and microvilli formation in the intestinal cells.

In mice, knocking out myosin Vb induces inclusions in enterocytes, reminiscent of the microvillus inclusion disease occurring upon myosin Vb mutation in human.^128^ In these mice, the inclusions are formed by bulk endocytosis of the brush border of the enterocytes. Strikingly, the inclusions were practically absent when the myosin Vb knockout mice were crossed with the PACSIN2 knockout mice, showing that PACSIN2 is involved in the formation of the inclusions.^128^ This study further supports the role for PACSIN2 in endocytic processes in the enterocytes.

### 6.3 *PACSIN2* polymorphism and gastrointestinal complications of thiopurines

In parallel to the insights provided by work on animal models, genetic studies have identified a potential role for *PACSIN2* polymorphism in the gastrointestinal toxicity caused by mercaptopurine.^129^, ^130^ Mercaptopurine belongs to the thiopurine family of drugs, and it is commonly used as a consolidation drug in the treatment of acute lymphoblastic leukaemia.^131^ Genetic polymorphisms in the enzyme thiopurine S‐methyltransferase (TPMT) are known to associate with mercaptopurine‐related toxicity. Moreover, a single nucleotide polymorphism in *PACSIN2* gene was found to associate with gastrointestinal toxicity of mercaptopurine.^129^ *PACSIN2* polymorphism was shown to associate with TPMT activity, and knocking down PACSIN2 in a leukemia cell line reduced TPMT activity.^129^ The effect of PACSIN2 polymorphism on TPMT activity is further supported by a study showing that PACSIN2 polymorphism associates with the 6‐thioguanine nucleosides/6‐methylmercaptopurine balance, controlled by TPMT and known to regulate the effectiveness and side effects of thiopurine.^132^, ^133^, ^134^ However, a recent study using a Taiwanese cohort found no association of PACSIN2 polymorphism with mercaptopurine toxicity.^135^ Moreover, the importance of *PACSIN2* polymorphisms in the complications of thiopurine therapies may be context specific as it was shown that PACSIN2 polymorphism does not associate with the adverse effect of thiopurine when used for the treatment of inflammatory bowel disease.^136^

## 7 PACSINS IN THE SENSORY SYSTEMS

### 7.1 Photoreceptor development and phototransduction in Drosophila and mammals

The eyes of *D. melanogaster* are composed of hundreds of functional units called ommatidia. Each ommatidium consists of light‐sensing neural cells, or photoreceptors, surrounded by non‐neural cells. The photoreceptors develop a photosensitive domain, the rhabdomere, which is composed of densely packed microvilli supported by a stalk, the rhabdomere‐supporting membrane. Syndapin localises at the base of the rhabdomeres in the neck region of the microvilli, at the interface between the apical domain of the cell and the rhabdomere itself.^137^ Syndapin mutant revealed that syndapin regulates the organization of the catacomb‐like membrane architecture of the apical domain of the rhabdomeres. The microvilli were dispersed and non‐parallel and the curved membrane at the base of the microvilli was lacking from the photoreceptors in the syndapin mutants compared to the wildtype flies.^137^ This indicates that the segregation of the stalk‐rhabdomere membrane is severely affected when syndapin is missing. Apparently, this membrane segregation function occurs through the membrane curving capacity of syndapin.^137^

In adult mice and rats, PACSIN1 is expressed in the retina.^46^, ^138^ The interaction of PACSIN1 with phosphodiesterase 6 subunit γ (PDE6γ) in rat retina homogenates suggests a possible role for PACSIN1 in phototransduction, as PDE6 is expressed at high levels in photoreceptors and is known to be a major effector of phototransduction in vertebrates.^46^, ^139^, ^140^ This is further supported by the presence of PACSIN1 around the synaptic ribbons in mice where it colocalizes with dynamin.^138^ Synaptic ribbons are parts of retinal ribbon synapses, which transmit sensory stimuli.^141^ The synaptic ribbons gather large amounts synaptic vesicles ready to be released and are thus active sites of both exo‐ and endocytosis.^141^ Similar to what is observed in the hippocampus, mice depleted of PACSIN1 present elevated synaptic vesicle size and enhanced number of endocytic structures.^68^ Since these structures are light‐sensitive, it will be interesting to further examine the role of PACSIN1 in vision as it appears that PACSINs plays a role in photosensitivity in both vertebrates and non‐vertebrates.

### 7.2 Retinal angiogenesis in mammals

In the developing mouse retina, PACSIN2 is involved in angiogenic sprouting or the generation of new blood vessels from existing ones.^142^ The authors found that on postnatal day 6, the retinas of PACSIN2 knockout mice have similar numbers of vascular branches and sprouts at the vascular front as the wild‐type controls, but the sprouts are shorter and display more cells at their base. This suggests a defect in the organization of leader and follower cells, the first ones migrating and the latter ones responsible for sprout elongation and vessel branching, in a process that relies on vascular endothelial (VE)‐cadherin ‐based adherens juntions.^142^, ^143^ There appeared more cytoplasmic VE‐cadherin in the sprouting front cells of the PACSIN2 knockout mouse retinas and the VE‐cadherin‐based junctions were disorganized.^142^ This is in line with previous in vitro work describing that PACSIN2 inhibits the internalization of VE‐cadherin at adherens junctions, suggesting a role for PACSIN2 in the maintenance of vascular integrity.^55^ Electric cell–substrate impedance sensing experiments further revealed that PACSIN2 knockdown cells have lowered capacity to form endothelial barriers. PACSIN2 knockdown in cultured primary human umbilical vein endothelial cells reduced the velocity of the healing front in wound healing assays and diminished sprout formation when the cells were cultured as spheroids in 3D matrices.^55^ Both in vitro and in vivo, endothelial cells depleted of PACSIN2 showed defective polarity.^55^, ^142^ The authors proposed that PACSIN2 drives the directionality of the angiogenic sprouting by regulating recycling events between the leader and follower cells in a mechanism involving EHD4 and MICAL‐L1,^142^ both known to regulate the formation of tubular recycling endosomes together with PACSIN2 in other cell models.^18^, ^21^ Specifically, EHD4 appears necessary for the recruitment of PACSIN2 to the asymmetric adherens junctions and for the adequate trafficking of VE‐cadherin, and thereby for the regulation of VE‐cadherin‐based adherence junctions involved in angiogenic sprouting.^142^ Overall, these studies suggest that PACSIN2 is involved in the development of retina by regulating endothelial barrier formation by a mechanism that involves regulation of the trafficking of VE‐cadherin by a PACSIN2‐EHD4‐MICAL‐L1 protein complex.^55^, ^142^

### 7.3 Laterality development and balance‐keeping in *D. rerio*

Morpholino‐mediated knockdown of PACSIN1 in *D. rerio* leads to laterality, or left‐right asymmetry defects, characterized by mislocalization of *pitx2*, a transcription factor known to regulate this process.^84^ Analysis of the swimming behaviour of the PACSIN1 MO‐treated fish reveals a loss of coordinated swimming and balance‐keeping capacity due to developmental alterations in the lateral line organ, which functionally resembles the inner ear in mammals. Specifically, PACSIN1 deficiency results in the reduction of the number of neuromasts, mechanosensory organs, and absence of cilia on their hair cells, responsible for sensing the waterflow around the fish. Interestingly, both actin‐rich stereocilia, which are non‐motile, sensory cilia and microtubule‐based kinocilia, which are motile cilia, were shorter in the absence of PACSIN1. Cobl MO‐treated *D. rerio* recapitulated the features of the PACSIN1 MO‐treated *D. rerio*, including the swimming, balance‐keeping and cilia formation defects. Co‐injection of reduced amounts of both PACSIN1 and Cobl MOs led to an even more severe phenotype, suggesting that as in mammalian cells, PACSIN1 and Cobl function together. This is supported by the colocalization of Cobl and PACSIN1 at the base of the cilia in culture and their presence in the same protein complex.^84^ Furthermore, rescue experiments of MO‐treated *D. rerio* revealed that both the membrane‐binding F‐BAR domain and Cobl‐recruiting SH3 domain of PACSIN1 are functionally necessary for PACSIN1‐mediated ciliogenesis.^84^

## 8 PACSIN2 IN THE KIDNEY

In the kidney, the expression of PACSIN2 varies with age and context. In the developing kidney, the ureteric bud gives rise to the collecting duct system via branching morphogenesis. Mesenchymal cells surrounding the tips of the branching ureteric tree undergo mesenchyme‐to‐epithelium transition and differentiate into nephrons, consisting of the glomerulus and the tubular part with proximal tubules, the loop of Henle and distal tubules. Throughout development and in the adult mice, PACSIN2 is expressed in the collecting ducts.^83^ In the new‐born mice in which nephrogenesis still continues in the kidney cortex, PACSIN2 is barely detectable in the early stages of nephron development but is expressed in the differentiated proximal and distal tubular segments of the nephron. In adulthood, the expression in the proximal tubule diminishes whereas it increases in the glomerulus and Bowman's capsule.^83^ Interestingly, induction of acute injury to the kidney by ischaemia‐reperfusion stimulates the expression of PACSIN2 in the proximal tubules and collecting ducts, suggesting a role for PACSIN2 in the tubular repair process in acute injury.^83^ The role of PACSIN2 in the repair process may rely on the capacity of PACSIN2 to regulate ciliogenesis, as in murine inner medullary collecting duct 3 cells, PACSIN2 regulates the length of primary cilia, the cilia being longer upon PACSIN2 knockdown. This, in turn, disturbs tubulogenesis in 3D culture models.^83^ The possible function of PACSINs in ciliogenesis is supported by work in *D. rerio*, showing that in the neuromasts the cilia are shorter upon PACSIN1 knockdown.^84^ A recent paper reported ciliogenesis defects also in *pacsin1b* and *pacsin2* mutant *D. rerio*, showing reduced cilia formation in otic vesicle or olfactory placode.^144^ Collectively, these data suggest a potential role for PACSIN2/syndapin in ciliary diseases, such as polycystic kidney disease.

In line with the above, a follow‐up study by Yao and colleagues revealed that PACSIN2 interacts with polycystin‐1,^47^ encoded by *PKD1*, which is mutated in autosomal dominant polycystic kidney disease.^145^ Interestingly, PACSIN2 was found to regulate the actin cytoskeleton together with polycystin‐1 and N‐WASP,^47^ a regulator of the actin cytoskeleton and an interaction partner of PACSINs.^5^ In the complex of these three proteins, PACSIN2 acts as an adaptor protein, which recruits N‐WASP, and together with polycystin‐1 activates N‐WASP. N‐WASP promotes actin nucleation, which could then regulate cell migration and the structure of the kidney tubules.^47^ Collectively, these data show that PACSIN2 is involved in the development and repair processes of kidney tubules apparently via its capacity to form multiprotein complexes that regulate actin remodeling and cell migration.

Recently, our laboratory found that PACSIN2 expression is increased in the glomeruli in the context of diabetic kidney disease.^23^ Specifically, we showed that PACSIN2 expression is elevated in the glomerular podocytes of obese and diabetic rats, and that this may perturb the intracellular trafficking of nephrin,^23^ a structural component of the cell junction that connects the adjacent podocytes.^146^ Nephrin, and specifically, its correct localization at the plasma membrane in the interpodocyte cell junction, is essential for kidney ultrafiltration.^147^, ^148^ Our results suggest that PACSIN2 regulates both internalization and recycling of nephrin, together with the rab4 and rab5 effector rabenosyn‐5.^23^ Interestingly, the association of PACSIN2 with nephrin is enhanced by either overexpression of rabenosyn‐5 or treating cultured podocytes with palmitate,^23^ the most abundant free‐fatty acid in humans and elevated in diabetes.^149^, ^150^

Overall, our studies and the ones by Yao and colleagues support a potential role for PACSIN2 in the kidney under pathological conditions, when adaptation of the intracellular trafficking and actin cytoskeleton organization may be needed to overcome stressful situations.^23^, ^47^, ^83^

## 9 PACSINS AND THE IMMUNE RESPONSE

A growing body of literature suggests a role for PACSINs in immune response. However, most of these studies are conducted in vitro, occasionally using ex vivo primary cultures. Here, we will summary just few of them as examples of the emerging importance of PACSINs in this field.

### 9.1 Immune response to viruses

In human, PACSIN1 is expressed in interferon‐producing plasmacytoid dendritic cells.^151^ These cells recognize pathogen‐derived DNA and RNA via toll‐like receptors 7 and 9 (TLR7 and TLR9). The authors compared the response of these immune cells, isolated from either PACSIN1 knockout or wildtype mice, after stimulation with influenza virus, vesicular stomatitis virus or herpes simplex virus 1. In all cases, the plasmacytoid dendritic cells deficient for PACSIN1 produced less interferon alpha than their wildtype counterparts, suggesting a role for PACSIN1 in the TLR7/9‐mediated response to viral stimulation. Interferon alpha production was also compromised in PACSIN1 deficient mice infected with herpes simplex virus 1.^151^

### 9.2 Spreading of viruses and bacteria

PACSIN2 is involved in viral dissemination of human immunodeficiency virus 1 (HIV‐1) and Rous sarcoma virus by associating with their Gag proteins.^40^ Gag proteins of HIV‐1 and other retroviruses participate in the hijacking of the intracellular machinery that is necessary for the release of virions.^152^ Interestingly, the authors show that PACSIN2 binds to ubiquitin‐Gag and is recruited to virus‐like particles in vitro. HIV‐1 spreading in T cell lines and in primary human mononuclear peripheral blood cells was prevented by the absence of PACSIN2, or when its SH3 domain was mutated and thus not capable of interacting with N‐WASP. This highlights the importance of PACSIN2 and its role in the regulation of actin polymerization for the dissemination of the HIV‐1 virus from cell to cell.^40^

Not only viruses, but also bacteria hijack PACSIN2‐dependent endosomal trafficking and actin machineries for cell‐to‐cell spread.^153^ PACSIN2, together with caveolin‐1 and −2, were found to regulate the cell‐to‐cell spread of *Listeria monocytogenes* in an RNAi screen.^153^ The spreading of *L. monocytogenes* includes generation of a double‐membrane protrusion extending from the donor cell into the recipient cell. In the recipient, the protrusion is surrounded by a vacuole with double‐membrane, and breakage of the vacuole then releases the bacteria into the recipient. Interestingly, knocking down PACSIN2, caveolin‐1 or caveolin‐2 results in reduced cell‐to‐cell spread of *L. monocytogenes*. Loss of these proteins impairs the efficacy of the recipient cell to engulf the protrusions, and the bacteria accumulate in the protrusions. Furthermore, refined analysis reveals that PACSIN2 is necessary in the recipient cell rather than in the donor cell, in line with its established role in membrane invaginations rather than protrusion.^153^

## 10 PACSINS IN THE NEUROENDOCRINE TISSUE

All PACSIN isoforms are expressed in the adrenal medulla in mice.^154^ Adrenal neuroendocrine cells, also called chromaffin cells, receive excitatory synaptic input from the sympathetic nervous system and transduce it by secreting hormones into the peripheral circulation.^155^ PACSINs are involved in the fusion pore expansion in the adrenal chromaffin cells, a key regulatory step that controls the release of the hormones.^65^, ^154^ Under stress when the sympathetic activity is high, dynamin I becomes dephosphorylated on serine 744, allowing it to bind to PACSINs, which in turn bind to and activate N‐WASP, leading to fusion pore expansion potentially via remodelling of the actin cytoskeleton.^65^ Disrupting the formation of this complex limits fusion pore expansion.^65^ The same research group revealed in a follow‐up study using isolated mouse primary chromaffin cells in culture that a mutation in the SH3 domain of PACSIN3 preventing its binding to dynamin 1, but not in PACSIN1 or PACSIN2, prevented the stress‐induced fusion pore expansion.^154^ This reveals a central role for PACSIN3 in the release of hormones from the adrenal chromaffin cells. However, more work will be required to define the involvement of PACSIN3 in the fusion pore expansion in vivo.

## 11 OMICS AND GENOME‐WIDE ASSOCIATION STUDIES

Several screening, omics and genome‐wide association studies have identified PACSIN family members to associate with human and animal diseases or traits.

### 11.1 PACSIN2 and platelet count

An interesting line of research highlights the potential role of PACSIN2 in platelet physiology. A large meta‐analysis aimed to identify the associations of single‐nucleotide variants with platelet count and mean platelet volume, the two key parameters used to screen and diagnose platelet‐associated disorders.^156^ The study identified an association of a common variant in ARFGAP3/PACSIN2 and decreased platelet count and increased mean platelet volume. Interestingly, PACSIN2 is abundant in platelets^157^ and has been shown to regulate membrane tubulation together with its binding partner filamin A in both megakaryocytes and platelets.^28^ This membrane tubulation activity is important when platelets are produced by megakaryocytes and when platelets spread after activation. Eicher et al (2016)^156^ proposed that the genetic variation in PACSIN2 could affect the membrane tabulation activity in megakaryocytes and thus lead to the production of a smaller amount of platelets that are larger in size.

### 11.2 PACSINs in cancer

PACSIN1 was highlighted in a study aiming to identify aberrant DNA methylation and variations in gene expression patterns that could be used as a prognostic tool to predict the progression of specific breast cancer subtypes.^158^ The study identified PACSIN1 as one of the eight signature genes, used to help to define the low‐risk and high‐risk groups, the latter having shorter survival time. The mRNA expression level of PACSIN1 was higher in the high‐risk group in comparison to the low‐risk group. The expression level of PACSIN1 was also higher in the aberrantly methylated groups compared to the control samples.^158^ However, the precise role of PACSIN proteins in cancer remains to be investigated.

### 11.3 PACSIN1 as a biomarker for idiopathic pulmonary fibrosis?

PACSIN1 mRNA was found to be differentially expressed in a study aiming to find novel biomarkers for idiopathic pulmonary fibrosis. Specifically, the expression of PACSIN1 was lower in the circulation of patients with severe compared to mild idiopathic pulmonary fibrosis.^159^ However, similar to the potential role of PACSIN1 in cancer onset and progression, the role for PACSIN1 in the development of pulmonary fibrosis remains to be confirmed by further studies.

### 11.4 PACSIN1 and risk of substance use

A genome‐wide, longitudinal study defined whether epigenetics, and specifically, DNA methylation, early in life is prospectively associated with substance use in adolescents.^160^ The study identified 65 differentially methylated loci at birth that prospectively associated with substance use in adolescents, with the most differentially methylated locus being that of PACSIN1. These loci were also shown to mediate, in part, the effects of tobacco smoking of pregnant mothers on the substance abuse by the children when teenagers. The identification of PACSIN1 as the most significant hit fits well with its localization in brain regions that are associated with drug‐seeking behaviour and addiction risk.^160^

### 11.5 PACSIN1 and body size

Polymorphisms in *PACSIN1* gene have been found to associate with body size or growth in several genetic and genome‐wide‐association studies, also coupled with meta‐analyses conducted in different pig populations.^161^, ^162^, ^163^, ^164^ The aim of these studies has been to identify genes or mutations that affect the development of muscles and other growth‐related factors in pigs to generate information for designing breeding strategies for meat production. The studies identify several single nucleotide polymorphisms in or near *PACSIN1* gene that associate with various traits related to the size of pigs, including loin muscle area and depth, body height, body length, cannon bone circumference, rump circumference and backfat thickness.^161^, ^162^, ^163^, ^164^ These studies support the idea that PACSIN1 may regulate the development or growth of muscle in mammals. However, further work is needed to identify the molecular pathways underlying the role of PACSIN1 in controlling the body size in pigs, and to define whether PACSIN1 regulates body size also in human.

## 12 CONCLUSIONS AND FUTURE DIRECTIONS

**FIGURE 4:**
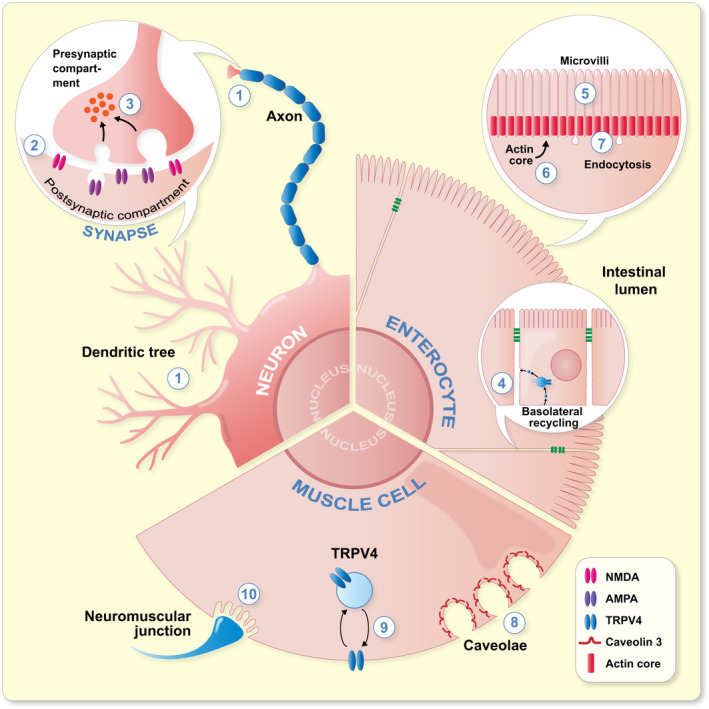
Specific functions of PACSINs in neurons, enterocytes and muscle cells. Neuron. 1: Neuromorphogenesis; regulation of the length and arborization of the dentritic tree and length of axons. 2: Synaptic plasticity; regulation of the trafficking of NMDA and AMPA receptors. 3: Synaptic vesicle endocytosis at the pre‐synaptic side. Enterocyte. 4: Basolateral recycling. 5: Formation and structure of the microvillae, coverage of the brush border by microvillae. 6: Localization of actin to the base of the microvillae and length of the actin core. 7: Endocytosis at the base of the microvillae. Muscle cell. 8: Formation of caveolae that act as membrane reservoirs. 9: Intracellular trafficking of TRPV4. 10: Formation of the subsynaptic reticulum on the muscular side of the neuromuscular junction

In summary, recent studies on animal models indicate that PACSINs are important for different physiological functions in various species across the whole tree of life, as summarized in Figure 3. First, PACSINs appear to be involved in specific steps of development of insects, fish, amphibians and mammals (Figure 3A). Knockout models also reveal that PACSINs play a role in maintaining the normal function of the brain, muscles and the digestive system (Figures 3B and 4). The importance of PACSINs in human diseases is suggested by genetic studies that revealed, for example, an association between PACSIN1 locus and schizophrenia. Furthermore, PACSINs may be involved in repair processes and the maintenance of organ physiology, as indicated by the upregulation of PACSIN2 in various kidney injury models.

Studies of PACSINs in specific organ systems show that disturbances in the well‐established functions of PACSINs, either in the regulation of intracellular trafficking or cytoskeleton organization, are responsible for the defects observed in the absence of PACSINs. Figure 4 illustrates some of the functions of PACSINs in specific cell types that have been extensively studied in vitro and show alterations in vivo when PACSINs are depleted. In neurons, for example, PACSIN1 regulates endocytosis of specific receptors via its membrane‐binding capacity, thereby modulating synaptic plasticity.^91^ PACSIN1 also regulates neuromorphogenesis by recruiting specific actin regulators to the plasma membrane.^91^ These defects, identified in vitro, likely result in the schizophrenia‐like behavior of PACSIN1 deficient mice.^91^ In enterocytes both in vitro and in vivo, PACSIN2 regulates the endocytic capacity and microvillar organization, which heavily relies on actin.^81^ This indicates that a finely tuned actin organization and endocytosis, both regulated by PACSIN2, are necessary for intestinal homeostasis.^81^ In the case of PACSIN3, the recent study on knockout mice indicates that in muscle cells, PASCIN3 is necessary for the formation of caveolae, which act as membrane reservoirs and preserve muscle physiology upon physical exercise.^17^ These studies highlight the importance of PACSINs in fundamental cellular processes and demonstrate that their absence results in the development of organ dysfunction. In many cases, the regulation of actin cytoskeleton and membrane trafficking processes by PACSINs are intertwined making it difficult to differentiate the significance of each function alone in vivo. Future studies will undoubtably further refine the exact mechanisms and participating molecular pathways of the cellular processes regulated by PACSINs.

Continuing the studies of the knockout models for PACSINs will without doubt reveal novel roles for PACSINs in developmental processes as well as provide more insights into the pathological events leading to various diseases. For example, while PACSIN2 depletion in mice does not lead to major vascular defects, PACSIN2 was found to regulate sprouting angiogenesis in the retina.^142^ In the case of pathological processes, several omics and genomic studies point out potential involvement of PACSINs in various diseases but await further work to validate the relevance of PACSINs in the pathological mechanisms associating with their development. The newly identified role of PACSIN2 in sprouting angiogenesis also raises an interesting question whether PACSIN2 could be involved in tumour vessel generation by regulating this process. In instances, PACSINs may be upregulated in disease processes, such as after acute tubular injury in the kidney, to participate in the repair process.^83^ Therefore, studying the phenotype of the mice lacking specific PACSINs may not reveal the significance of PACSINs without additional challenges or disease induction. For example, inducing diabetes in PACSIN2 knockout mice will reveal whether PACSIN2 is involved in the regulation of the renal function under diabetic conditions. More work is also needed to confirm the extend of the role of PACSIN1 in human neurological disorders, as suggested based on work using PACSIN1 knockout mice^68^, ^91^ and genetic studies. Ultimately PACSINs, or the pathways regulated by PACSINs, may prove to be plausible targets for developing new therapeutic approaches for various diseases affecting the organ systems.

## CONFLICT OF INTEREST

The authors declare no conflicts of interests.
